# A phase II clinical trial of neoadjuvant chemotherapy combined with immunotherapy and different radiotherapy fractionation regimens in HR+/HER2- breast cancer

**DOI:** 10.3389/fimmu.2026.1836220

**Published:** 2026-07-09

**Authors:** Jie Lan, Xiaoyue Sun, Xiaobo Huang, Yanxia Zhao, Zhuofei Bi, Wei Huang, Tin Luo, Jing Jing, Xin Wu, Lei Liu

**Affiliations:** 1 Institute of Breast Health Medicine, State Key Laboratory of Biotherapy, West China Hospital, Sichuan University and Collaborative Innovation Center, Chengdu, China; 2 Division of Head and Neck Tumor Multimodality Treatment, Cancer Center, West China Hospital, Sichuan University, Chengdu, China; 3 Department of Radiotherapy for Breast Cancer, Yat-sen Breast Tumor Hospital, Sun Yat-sen Memorial Hospital, Sun Yat-sen University, Guangzhou, China; 4 Guangdong Provincial Key Laboratory of Malignant Tumor Epigenetics and Gene Regulation, Medical Research Center, Sun Yat-sen Memorial Hospital, Sun Yat-sen University, Guangzhou, China; 5 Cancer Center, Union Hospital, Tongji Medical College, Huazhong University of Science and Technology, Wuhan, China; 6 Hubei Key Laboratory of Precision Radiation Oncology, Tongji Medical College, Huazhong University of Science and Technology, Wuhan, China; 7 Department of Radiation Oncology, Shandong Cancer Hospital and Institute, Shandong First Medical University, Shandong Academy of Medical Sciences, Jinan, China

**Keywords:** breast cancer, chemotherapy, immunotherapy, neoadjuvant therapy, radiotherapy

## Abstract

**Background:**

The modest neoadjuvant response in early-stage HR+/HER2- breast cancer necessitates novel treatment intensification strategies. While radiotherapy can enhance systemic efficacy via immune modulation, the optimal fractionation for integration with immunochemotherapy remains unknown. This multicenter phase II study aims to evaluate the feasibility, safety, and preliminary efficacy of Toripalimab combined with chemotherapy and tumor-directed radiotherapy, specifically investigating three distinct fractionation schedules.

**Methods:**

This prospective, multicenter, three-cohort exploratory study enrolls treatment-naïve patients with early-stage HR+/HER2- breast cancer. Participants receive neoadjuvant Toripalimab plus chemotherapy alongside image-guided radiotherapy restricted to the primary tumor. Cohorts are differentiated by fractionation regimen: Arm 1 (8 Gy × 3 fractions, total 24 Gy); Arm 2 (16 Gy single fraction); Arm 3 (0.5 Gy twice daily for 8 cycles, cumulative 8 Gy). A total of 45 patients (15 per cohort) will be enrolled. The primary endpoint is pathological complete response (pCR). Secondary endpoints include objective response rate, recurrence, survival outcomes (OS, EFS, DMFS, IDFS, IBTR), and safety.

**Conclusions:**

This exploratory study will provide feasibility, tolerability, and preliminary efficacy data for a novel multimodal neoadjuvant strategy combining different radiotherapy fractionation patterns with immunotherapy and chemotherapy. Given the descriptive analytical framework, findings are expected to be hypothesis generating and will inform the selection of radiotherapy schedules for future larger-scale randomized trials aimed at improving outcomes in HR+/HER2- breast cancer.

**Clinical trial registration:**

http://www.chictr.org.cn, identifier NCT06639672.

## 1 Introduction

Breast cancer remains the most commonly diagnosed malignancy among women worldwide, with over 2.3 million new cases and approximately 685,000 deaths annually, representing a substantial global public health burden (1).

Hormone receptor positive/human epidermal growth factor receptor 2 negative (HR+/HER2-) breast cancer is the most prevalent subtype. Although generally associated with a more favorable long-term prognosis compared with other subtypes, HR+/HER2- tumors demonstrate limited sensitivity to neoadjuvant systemic therapy, with relatively low pathological complete response (pCR) rates (2, 3). Given the established association between pCR and improved long-term outcomes in high-risk early breast cancer, strategies to enhance neoadjuvant efficacy in HR+/HER2- disease represent an important unmet clinical need (4).

Immune checkpoint inhibitors (ICIs), particularly PD-1/PD-L1 inhibitors, have revolutionized the treatment of multiple malignancies (5–7). In breast cancer, early evidence suggested limited immunogenicity, particularly in HR+/HER2- tumors, which are often characterized as immunologically “cold.” However, recent phase III trials have demonstrated that adding ICIs to chemotherapy can significantly improve pCR rates in high-risk HR+/HER2- breast cancer. In CheckMate 7FL and KEYNOTE-756, neoadjuvant immunochemotherapy increased pCR rates by approximately 8-11% compared with chemotherapy alone (8, 9). While these findings establish proof of concept, absolute pCR rates remain modest, underscoring the need for further therapeutic optimization.

Although radiotherapy is traditionally regarded as a locoregional treatment for breast cancer, accumulating evidence shows that it can induce immunogenic cell death, releasing tumor antigens and promoting dendritic cell maturation, which in turn facilitates T-cell infiltration and creates an *in situ* vaccination-like effect (10–13). These immunological changes can convert previously quiescent tumors into inflamed, immune-responsive phenotypes, thereby sensitizing them to immune checkpoint blockade and overcoming primary resistance (14–16). Collectively, these findings provide a strong biological rationale for combining radiotherapy with immune checkpoint inhibitors.

Given the immunomodulatory properties of radiotherapy, its combination with immune checkpoint blockade offers a promising strategy to improve the modest response of breast cancer to immunotherapy alone (17). The randomized phase II Neo-CheckRay trial evaluated neoadjuvant Durvalumab (with or without Oleclumab) plus chemotherapy and hypofractionated radiotherapy (8 Gy×3 fractions) in high-risk HR+/HER2- breast cancer. Primary endpoint results presented at ESMO 2024 showed higher RCB 0/1 and pCR rates with the addition of immunotherapy (18). The subsequent full publication supported these preliminary findings, showing numerically higher RCB 0/1 and pCR rates in the immunotherapy-containing arms, with increased grade ≥3 treatment-related adverse events but no deaths or grade ≥3 iSBRT-related toxicities, supporting the overall feasibility of this approach (19). Collectively, these findings warrant further clinical investigation of the safety and antitumor activity of this neoadjuvant combination.

Building on these data, the present study investigates a novel neoadjuvant strategy integrating radiotherapy with chemotherapy and PD-1 blockade in early-stage HR+/HER2- breast cancer. In this trial, Toripalimab is administered in combination with chemotherapy consisting of nab‑paclitaxel, followed by epirubicin plus cyclophosphamide, along with concurrent modified hypofractionated radiotherapy to the primary tumor. Distinct from conventional comprehensive field irradiation, this tumor-focused approach with tailored dose fractionation is designed not only to minimize unnecessary normal tissue exposure and improve tolerability, but more importantly, to harness the immunogenic effects of radiation, enhancing *in situ* tumor antigen release and T-cell priming within the context of PD-1 inhibition. To explore divergent radiobiological strategies, three fractionation schedules are incorporated: moderate hypofractionation (8 Gy×3 fractions or 16 Gy single fraction) and an ultra-low-dose, ultra-fractionated regimen (0.5 Gy twice daily). Given the limited responsiveness of this subtype to existing neoadjuvant regimens, this trial seeks to evaluate the safety, feasibility, and preliminary efficacy of this multimodal combination and to generate hypothesis generating data to inform the design of future larger-scale randomized studies.

## 2 Methods/design

### 2.1 Study design

This study is a multicenter, multicohort, prospective phase II clinical trial that will enroll participants at four institutions: West China Hospital of Sichuan University, Shandong Cancer Hospital, Sun Yat-sen Memorial Hospital of Sun Yat-sen University, and Union Hospital affiliated with Huazhong University of Science and Technology.

After confirmation of eligibility, participants will be randomly assigned in a 1:1:1 ratio to one of three intervention arms:

Arm 1: radiotherapy 24 Gy in 3 fractions.
Arm 2: radiotherapy 16 Gy in a single fraction.
Arm 3: radiotherapy 0.5 Gy twice daily on days 1of every cycle with an interval of at least 6 hours between fractions, for a total of 8 cycles, resulting in a cumulative dose of 8 Gy.

### 2.2 Objectives and endpoints

#### 2.2.1 Primary endpoint

The primary endpoint of the study is pCR, assessed after completion of neoadjuvant therapy and surgery. pCR is defined as the absence of residual invasive tumor cells in both the breast and axillary lymph nodes.

#### 2.2.2 Secondary endpoints

Secondary efficacy endpoints include: objective response rate (ORR) according to RECIST version 1.1, overall survival (OS), event-free survival (EFS), ipsilateral breast tumor recurrence (IBTR), distant metastasis-free survival (DMFS), and invasive disease-free survival (IDFS).

#### 2.2.3 Safety endpoints

Safety endpoints will include the incidence, severity, and spectrum of adverse events (AEs), including serious adverse events (SAEs), immune-related adverse events (irAEs), and radiation-associated toxicities. Adverse events will be systematically collected from treatment initiation through the predefined safety follow-up period and graded according to the National Cancer Institute Common Terminology Criteria for Adverse Events (CTCAE), version 5.0. Radiotherapy-related toxicities will additionally be evaluated using established RTOG radiation toxicity criteria.

#### 2.2.4 Tumor assessment

Tumor response will be assessed radiologically according to RECIST version 1.1, using magnetic resonance imaging (MRI) and ultrasonography. The first imaging assessment will be conducted 6 weeks after the first dose of study treatment. Thereafter, tumor assessments will be performed every 6 weeks ± 7 days during neoadjuvant treatment and every 12 weeks after surgery, or as clinically indicated, until radiographically confirmed disease progression, initiation of a new antitumor therapy, death, or withdrawal of informed consent, whichever occurs first.

### 2.3 Study population

#### 2.3.1 Inclusion criteria

- Female patients aged 18 to 65 years, with a body mass index (BMI) ≥18.
- Pathologically confirmed HR+/HER2- breast cancer, defined as estrogen receptor positive, progesterone receptor negative or positive, and HER2 negative, or HER2 1+ or 2+ by immunohistochemistry with negative fluorescence *in situ* hybridization.
- Previously untreated disease without evidence of distant metastasis.
- Clinical stage cT1cN1 to N2M0 or cT2N0 to N2M0 according to the American Joint Committee on Cancer (AJCC) eighth edition staging system.
- Eastern Cooperative Oncology Group performance status (ECOG PS) score of 0 or 1.
- Baseline left ventricular ejection fraction ≥55% as assessed by echocardiography, preferred.
- Adequate bone marrow function, defined as: white blood cell count ≥3.5 × 10^9/L, absolute neutrophil count ≥1.5 × 10^9/L, platelet count ≥100 × 10^9/L, and hemoglobin ≥90 g/L.
- Adequate hepatic function, defined as: total bilirubin ≤1.5 times the upper limit of normal, aspartate aminotransferase and alanine aminotransferase ≤2.5 times the upper limit of normal.
- Adequate renal function, defined as: serum creatinine ≤1.5 times the upper limit of normal, or calculated creatinine clearance ≥60 ml/min using the Cockcroft Gault formula.
- Adequate coagulation function, defined as an international normalized ratio or prothrombin time ≤1.5 times the upper limit of normal. For patients receiving anticoagulant therapy, prothrombin time must be within the intended therapeutic range of the anticoagulant.
- Women of childbearing potential must agree to use effective contraception from the time of signing informed consent until 180 days after the last dose of study treatment. Women of childbearing potential include premenopausal women and women within two years after menopause. A serum pregnancy test within three days before the first administration of the study drug must be negative.
- No severe organic heart disease or clinically significant arrhythmia.
- Provision of written informed consent by the patient.

#### 2.3.2 Exclusion criteria

- Patients with communication or comprehension impairment that precludes reliable participation or informed responses, including conditions such as impaired consciousness, aphasia, or intellectual disability.
- Poorly controlled tumor-related pain. Patients requiring analgesics must have a stable pain control regimen at the time of study entry.
- Presence of active or prior autoimmune disease or immunodeficiency, including but not limited to myasthenia gravis, myositis, autoimmune hepatitis, systemic lupus erythematosus, rheumatoid arthritis, inflammatory bowel disease, antiphospholipid syndrome, Wegener granulomatosis, Sjögren syndrome, Guillain-Barré syndrome, or multiple sclerosis.
- Patients with a history of autoimmune hypothyroidism who are receiving thyroid hormone replacement therapy are eligible for this study.
- Patients with type 1 diabetes mellitus who are receiving insulin therapy and achieving adequate glycemic control are eligible for this study.
- Patients with eczema, psoriasis, chronic simple lichen, or vitiligo limited to cutaneous involvement only, for example, excluding psoriatic arthritis, may be eligible provided that all of the following conditions are met: The affected skin area must involve less than 10% of the body surface area. The disease must be well controlled at baseline and require only topical low-potency corticosteroids. There must have been no acute exacerbation requiring systemic therapy within the past 12 months, including psoralen plus ultraviolet A, methotrexate, retinoids, biologic agents, oral calcineurin inhibitors, or high-potency or oral corticosteroids.
- History of idiopathic pulmonary fibrosis, organizing pneumonia such as bronchiolitis obliterans, drug-induced pneumonitis, or idiopathic pneumonitis, or evidence of active pneumonitis on screening chest computed tomography. Patients with a history of radiation pneumonitis or pulmonary fibrosis within the radiation field are excluded.
- Active pulmonary tuberculosis.
- Serious cardiovascular disease within three months before initiation of study treatment, including but not limited to New York Heart Association class II or higher heart failure, myocardial infarction, or cerebrovascular accident, as well as unstable arrhythmia or unstable angina.
- Major surgical procedure other than diagnostic surgery within four weeks before initiation of study treatment, or anticipated need for major surgery during the study period.
- History of malignancy other than breast cancer within five years before study entry, except for malignancies with negligible risk of metastasis or death, such as adequately treated cervical carcinoma *in situ*, non-melanoma skin cancer, ductal carcinoma *in situ*, or stage I uterine cancer.
- Severe infection within four weeks before initiation of study treatment, including but not limited to hospitalization for infection, bacteremia, severe pneumonia, or any active infection that may compromise patient safety.
- Prior allogeneic hematopoietic stem cell transplantation or solid organ transplantation.
- Any other disease, metabolic disorder, abnormal physical finding, or laboratory abnormality that is a contraindication to study drugs may confound the interpretation of study results or may place the patient at unacceptable risk.
- Receipt of a live attenuated vaccine within four weeks before initiation of study treatment, or anticipated need for such vaccination during Toripalimab treatment, or within five months after the last dose of Toripalimab.
- Active hepatitis B infection, defined as hepatitis B surface antigen positivity at baseline with hepatitis B virus DNA level above the upper limit of normal at the local laboratory.
- Prior treatment with CD137 agonists or immune checkpoint blockade, including therapeutic antibodies against CTLA-4, PD-1, or PD-L1.
- Receipt of systemic immune stimulating agents within four weeks or five drug elimination half-lives, whichever is longer, before initiation of study treatment, including but not limited to interferons and interleukin 2.
- Use of systemic immunosuppressive medications within two weeks before initiation of study treatment.
- History of severe hypersensitivity to chimeric or humanized antibodies or fusion proteins.
- Known hypersensitivity to Chinese hamster ovary cell-derived products or to any component of the Toripalimab formulation.
- Known allergy or hypersensitivity to any component of Nab-paclitaxel, Epirubicin, or Cyclophosphamide.

Any other condition that, in the opinion of the investigator, would make the patient unsuitable for study participation or interfere with completion of the study.

### 2.4 Description of interventions

**Figure 1:**
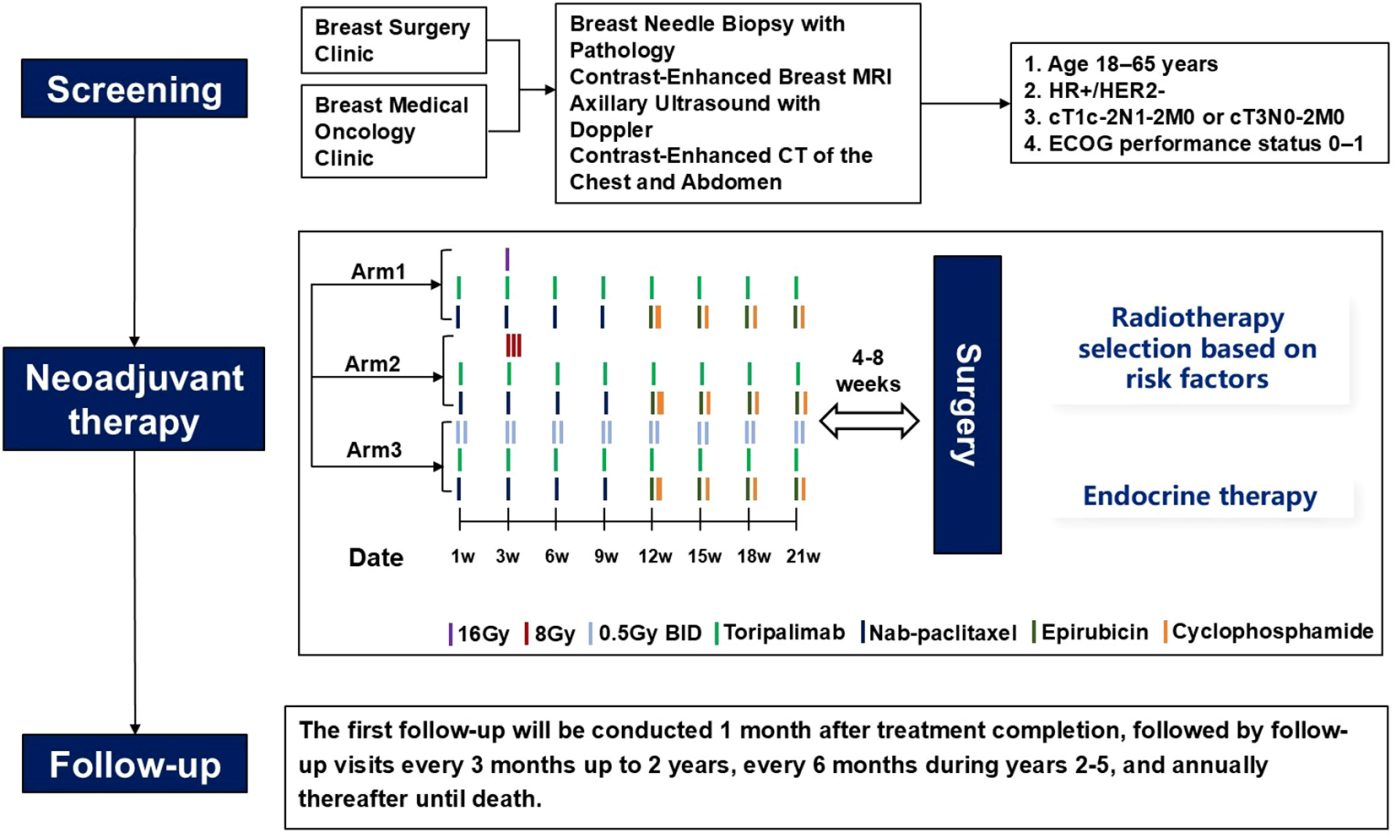
Study treatments of the phase II randomized trial.

This study consists of three treatment arms. Different radiotherapy fractionation regimens will be administered in combination with chemotherapy and immunotherapy as neoadjuvant treatment. Surgery will be scheduled 4–8 weeks after completion of the final cycle of neoadjuvant therapy (**Figure 1**).

#### 2.4.1 Neoadjuvant radiotherapy

Radiotherapy will be delivered using 6 MV X-rays under CT/MRI image guidance with intensity-modulated radiotherapy (IMRT).

##### 2.4.1.1 Dose specifications

Arm 1: 8 Gy × 3 fractions, administered every other day; irradiation limited to the primary tumor lesion.

Arm 2: 16 Gy × 1 fraction; irradiation limited to the primary tumor lesion.

Arm 3: 0.5 Gy twice daily on day 1 of every cycle with an interval of at least 6 hours between fractions, resulting in a cumulative dose of 8 Gy; irradiation limited to the primary tumor lesion.

##### 2.4.1.2 Dose prescription deviations

Radiotherapy interruptions should be avoided, except for scheduled holidays or equipment malfunctions. Interruptions are permitted only for febrile neutropenia, or Grade ≥3 esophagitis/mucositis, skin toxicity, or pulmonary toxicity. All interruptions must be documented in the Case Report Form (CRF). Missed radiotherapy fractions must not be compensated by dose doubling or twice-daily administration in a single day. The overall frequency and specific dates of missed fractions must be carefully recorded.

##### 2.4.1.3 Deviation definitions

- No deviation: ≥95% of the planning gross tumor volume (PGTV) receives ≥95% of the prescribed dose, and no region outside the PGTV receives >110% of the prescribed dose.
- Minor deviation: ≥95% of the PGTV receives ≥93% of the prescribed dose, or adjacent non-PGTV regions receive >110% but ≤115% of the prescribed dose.
- Major deviation: <95% of the PGTV receives ≥93% of the prescribed dose, or adjacent non-PGTV regions receive >115% of the prescribed dose.

Major deviations are generally considered unacceptable.

##### 2.4.1.4 Positioning, simulation, and immobilization

Participants are immobilized supine using standard devices, with the same device used for simulation and treatment. Contrast-enhanced CT and MRI (3 mm slices, quiet breathing) are acquired from the mandible to the inferior border of L2 for simulation and co-registration. This scan range supports dose calculation but does not define the treatment field, which is confined to the tumor region with predefined margins.

##### 2.4.1.5 Treatment planning and target volumes

Target delineation is based on fused CT/MRI. GTV is defined as the visible primary tumor, and PGTV is derived from the GTV with an isotropic margin of 0.3 cm to account for motion and setup uncertainties. Planning uses volumetric dose optimization, and DVHs are generated for the target and the organs at risk (OARs) (lungs, spinal cord, heart, skin, chest wall).

##### 2.4.1.6 OARs

Normal tissue constraints must be prioritized in the following order:

Spinal cord > Lung > Heart > Skin > Chest wall.

Spinal Cord: The spinal canal contour will represent the spinal cord. The maximum dose (Dmax) must not exceed 3 Gy.

Lung: Lung dose constraints rank second in priority and must be satisfied unless conflicting with spinal cord constraints. V10 ≤ 20%.

Heart: The entire heart should be contoured, including the right ventricular outflow tract and the apices of both atria (excluding major vessels where feasible) to the inferior border of the left ventricle.

For Left-sided breast cancer: Mean heart dose (Dmean) ≤ 200 cGy; V5 ≤ 10%.

For Right-sided breast cancer: Dmean ≤ 200 cGy; V5 ≤ 10%.

Skin: Contoured within ±3 cm superior and inferior to the PGTV, including subcutaneous tissue to a depth of 0.5 cm. Dmax < 2500 cGy, V1500 cGy < 10 cm³.

Chest Wall: Contoured within ±3 cm superior and inferior to the PGTV, including ribs and intercostal muscles. V1500 cGy < 3 cm³.

##### 2.4.1.7 Radiotherapy quality assurance (RTQA)

To ensure consistency across centers, a dedicated RTQA program will be implemented in this study. The program covers site credentialing, standardized procedures for simulation and target delineation, image−guided verification requirements, and deviation documentation. All radiotherapy−related deviations will be categorized and reported per protocol.

#### 2.4.2 Neoadjuvant chemotherapy

Nab-paclitaxel: 260 mg/m², intravenous (IV), Day 1, every 3 weeks (Q3W), for the first 4 cycles.

Epirubicin: 90–100 mg/m², IV, Day 1, Q3W, for the subsequent 4 cycles.

Cyclophosphamide: 600 mg/m², IV, Day 1, Q3W, or 900 mg/m² orally, with the total dose evenly divided over days 1-14, Q3W, for the last 4 cycles.

#### 2.4.3 Neoadjuvant immunotherapy

Toripalimab: 240 mg IV, Day 1, Q3W, for 8 cycles.

#### 2.4.4 Surgery

Surgery is scheduled 4–8 weeks after neoadjuvant therapy. The surgical approach, either breast-conserving surgery or mastectomy, will be determined by both the extent of tumor involvement and the patient’s preference. Sentinel lymph node biopsy (SLNB) or axillary lymph node dissection (ALND) will be performed based on pre-neoadjuvant pathological findings and post-neoadjuvant axillary lymph node status.

#### 2.4.5 Postoperative adjuvant therapy

Patients with HR+ disease will routinely receive adjuvant endocrine therapy. Postoperative adjuvant chemotherapy or radiotherapy will be determined according to postoperative pathological findings.

### 2.5 Procedures and data collection

#### 2.5.1 Screening and baseline assessment (day -28 to day -1)

Informed consent and ID: Obtain written informed consent; assign a unique study ID.

Baseline data: Collect demographics, medical history, prior treatments; record vital signs.

Laboratory and ancillary tests: complete blood count (CBC), serum biochemistry (liver/renal function), coagulation, tumor markers (carcinoembryonic antigen, cancer antigen 125, cancer antigen 153), thyroid function, cardiac enzymes (creatine kinase MB isoenzyme, B-type natriuretic peptide, cardiac troponin), electrocardiography (ECG); transthoracic echocardiography(TTE); infectious disease screening: human immunodeficiency virus (HIV) antibody test, hepatitis B virus (HBV) serologic markers (HBsAg, HBsAb, HBcAb, HBeAg, HBeAb) and HBV DNA, hepatitis C virus (HCV) antibody and HCV RNA, and and pregnancy test (if applicable).

Imaging: Obtain contrast/non-contrast CT or MRI of head, neck, chest, and whole abdomen; breast MRI and dedicated breast ultrasound; whole body bone scan.

Lesion marking: Mark the primary lesion with at least two reference points along the longest diameter to monitor neoadjuvant therapy response.

Enrollment confirmation: Review all results, confirm eligibility, and schedule the first treatment appointment.

#### 2.5.2 Neoadjuvant treatment period

Day 0 assessment: On the day prior to Cycle 1, confirm fitness via clinical and laboratory evaluation. Complete simulation and treatment planning.

Radiotherapy regimens (delivered during Cycle 2, except Arm 3).

- Arm 1: 8 Gy on Days 1, 3, 5 (total 24 Gy).
- Arm 2: 16 Gy on Day 1 (single fraction, total 16 Gy).
- Arm 3: 0.5 Gy twice daily on Day 1 of each of 8 cycles (total 8 Gy).

Chemotherapy (every 3 weeks, Day 1): Toripalimab 240 mg IV, Nab-paclitaxel 260 mg/m² IV or Epirubicin 90–100 mg/m² IV and Cyclophosphamide 600 mg/m² IV or 900 mg/m² oral divided over Days 1-14.

Safety monitoring: Monitor Adverse Events (AEs); perform protocol-specified labs (CBC, biochemistry, coagulation, tumor markers, thyroid, cardiac enzymes) during and after treatment.

Cycle continuation: Review AEs after each cycle; proceed if no SAEs or discontinuation criteria are met.

Post−Cycle 8 evaluation (within 4 weeks): Thyroid ultrasound, T, CT/MRI of head/neck/chest/abdomen (PET/CT optional); bone scan only if not done at screening. Assess tumor response per RECIST v1.1 before surgery.

Tumor response follow−up: First imaging assessment 6 weeks after the first treatment, including breast MRI and breast and axillary ultrasound; every 6 weeks ± 7 days neoadjuvant treatment and every 12 weeks after surgery, or as clinically indicated, until radiologically confirmed disease progression, initiation of new anti-tumor therapy, death, or withdrawal of informed consent, whichever occurs first.

#### 2.5.3 Perioperative period (4–8 weeks after neoadjuvant therapy)

Surgery is scheduled 4–8 weeks after completing neoadjuvant therapy. The type of surgery (breast-conserving or mastectomy) is selected based on tumor extent, while axillary management (SLNB or ALND) is determined by baseline pathology and post-treatment nodal status. Preoperatively, routine assessments (including laboratory tests and ECG) are performed to confirm fitness for surgery. Postoperatively, vital signs and surgical site complications (e.g., bleeding, infection, wound dehiscence) are closely monitored and managed as needed.

#### 2.5.4 Postoperative adjuvant therapy period

Adjuvant endocrine therapy is routinely given to hormone receptor−positive (HR+) patients. Adjuvant chemotherapy or radiotherapy after surgery is assessed according to the patient’s preoperative stage and postoperative pathological status.

#### 2.5.5 Follow-up period

Follow-up starts after surgery or treatment discontinuation. The first visit at 30 days post−surgery includes physical examination, vital signs, ECOG performance status, CBC, urinalysis, serum biochemistry, and coagulation profile. Subsequent visits occur every 3 months for 2 years.

### 2.6 Specimen collection and handling procedures

Tumor tissue (collected at baseline biopsy and/or surgery) and peripheral blood (drawn at predefined time points during treatment and follow-up) are collected for project-specific research, which includes assessment of pathological response, treatment-related toxicity, and exploratory biomarkers. Blood samples are processed within 2 hours, and serum/plasma are stored at -80°C. Routine safety labs are performed locally. All specimens are coded with unique identifiers to ensure traceability.

### 2.7 Statistical analysis

This exploratory study enrolls 45 patients (15 per cohort) and is not powered for formal hypothesis testing; all analyses are descriptive. Efficacy analyses use Intention-to-Treat; safety analyses use the treated population. Categorical endpoints (pCR, ORR) are reported with frequencies and 95% Clopper-Pearson CIs; continuous variables with mean, standard deviation, median, and range. Time to event outcomes (OS, EFS, IBTR/LRR, DMFS, IDFS) are estimated by Kaplan-Meier with 95% CIs (interpreted cautiously given small cohorts). AEs are graded per CTCAE v5.0, for repeated AEs, the highest grade per participant is used. Safety summaries include all AEs, SAEs, Treatment-Related Adverse Events, and discontinuations. Deaths are tabulated.

### 2.8 Ethical considerations and data protection

This study is conducted in compliance with the Declaration of Helsinki, International Council for Harmonisation Good Clinical Practice (ICH-GCP), and applicable national regulations. The protocol and related documents are reviewed and approved by the Institutional Ethics Committee prior to initiation. All participants provide written informed consent before any study-specific procedures and may withdraw at any time without prejudice to their future care. Although the treatment involves risks of chemotherapy-related, immune-related, and radiation toxicities, the potential benefit of improved tumor response is considered to outweigh these risks based on current evidence, making the overall benefit−risk profile acceptable.

### 2.9 Data management

Clinical data will be recorded in case report forms (CRFs) by trained study personnel and entered into a secure electronic database. To ensure accuracy and completeness, regular data verification and source document review will be performed. All study documents and essential records will be retained for the period required by applicable regulatory authorities.

### 2.10 Clinical data monitoring

Clinical monitoring will be performed to ensure participant safety, protocol adherence, and data integrity. Monitoring procedures will include verification of informed consent, eligibility confirmation, source data verification, and review of adverse event reporting. Serious adverse events will be reported according to regulatory requirements. The sponsor reserves the right to suspend enrollment if unacceptable safety concerns emerge.

### 2.11 Protocol deviations

All protocol deviations will be documented and categorized according to their potential impact on patient safety and study integrity. Major deviations include violation of eligibility criteria, significant radiotherapy dose deviations beyond predefined thresholds, failure to deliver assigned treatment, or incomplete primary endpoint assessment. Radiotherapy plan deviations will be classified according to predefined target coverage and organ-at-risk constraints. Deviations affecting participant safety or data validity will be reported to the ethics committee in accordance with institutional policies.

## 3 Discussion

Neoadjuvant management of HR+/HER2- breast cancer remains clinically challenging, particularly in patients with biologically high-risk disease (20). Although current systemic strategies yield measurable tumor shrinkage, the overall response in this subtype is generally modest, with most patients failing to achieve sufficient downstaging to permit surgical deescalation (4, 21). Enhancing early treatment efficacy therefore represents a critical unmet need in this population.

To address this limitation, the present study investigates a multimodal regimen that integrates tumor directed radiotherapy into an immunochemotherapy backbone. The biological rationale for this strategy is supported by the immunomodulatory properties of radiotherapy. Preclinical and clinical evidence suggests that radiation can induce immunogenic cell death, release tumor associated antigens, and promote dendritic cell maturation, thereby enhancing T cell priming and infiltration effects that may convert immunologically “cold” HR+/HER2- tumors into more inflamed phenotypes (10–14, 22, 23). This *in situ* vaccination effect provides a strong rationale for combining localized radiotherapy with PD-1 blockade and chemotherapy in the neoadjuvant setting.

Three distinct radiotherapy fractionation schedules were selected to represent divergent radiobiological strategies for leveraging radiation−induced immune effects. Arm 1 (8 Gy×3 fractions, total 24 Gy) and Arm 2 (16 Gy single fraction) both employ moderate hypofractionation, which has been associated with enhanced type I interferon signaling and CD8+ T cell cross priming (14). However, Arm 2 delivers a higher single dose exposure that may maximize direct cytotoxicity and danger associated molecular pattern signaling, although whether single fraction radiotherapy offers superior immune activation over multifraction regimens remains debated. Arm 3 (0.5 Gy twice daily for 8 cycles, cumulative 8 Gy) explores an ultra-low-dose, ultra-fractionated approach hypothesized to favor preservation of lymphoid compartments, reduce immunosuppressive signaling, and allow repeated, sustained antigen exposure over time (24). While the optimal fractionation for immune activation is not yet defined, this cohort design enables preliminary comparison of feasibility, safety, and antitumor activity across regimens, and the findings will inform the selection of fractionation schedules for future larger-scale trials.

This study investigates a multimodal approach that integrates tumor-directed radiotherapy into an immunochemotherapy backbone. Rather than adopting conventional wide-field radiation paradigms, the protocol employs focused irradiation and alternative fractionation schedules with the intent of balancing immune activation and treatment tolerability. By exploring distinct radiotherapy regimens within a structured cohort design, the study aims to generate clinically relevant data regarding feasibility and safety in this setting.

Toripalimab is administered at 240 mg intravenously every 3 weeks, a dose selected based on prior phase I-III studies demonstrating consistent pharmacokinetic exposure and antitumor activity across multiple solid tumor indications. The radiotherapy technique employed is IMRT under CT/MRI image guidance, with detailed target delineation and ORA constraints described in the Methods section (2.4.1).

Comparing our design with existing evidence, the randomized phase II Neo-CheckRay trial recently evaluated neoadjuvant Durvalumab (with or without Oleclumab) plus chemotherapy and hypofractionated radiotherapy (24 Gy/3 fractions) in high-risk HR+/HER2- breast cancer. Primary endpoint results showed higher rates of RCB 0/1 and pCR with immunotherapy-containing arms (18, 19). While Neo-CheckRay provides important proof-of-concept, our study extends this by systematically evaluating multiple radiotherapy schedules within the same immunochemotherapy backbone, specifically including a novel ultra-low-dose regimen and by using Toripalimab, a PD-1 inhibitor with established efficacy in several solid tumors.

From a practical standpoint, strategies that improve early response may expand eligibility for breast-conserving procedures, facilitate surgical planning, and potentially reduce the burden of residual disease (25, 26). Given the high prevalence of this breast cancer subtype, even incremental improvements in response could have meaningful implications for healthcare systems, particularly in settings where recurrence management imposes substantial long-term costs (27).

Several limitations of this exploratory phase II study should be acknowledged. First, the sample size (15 patients per cohort, total 45) is not powered for formal hypothesis testing; all inter cohort comparisons will be descriptive and hypothesis generating only. Second, the absence of predefined translational biomarker or immune correlative analyses limits the ability to definitively attribute observed responses to radiotherapy induced immune modulation versus chemotherapy or immunotherapy effects. Pathological response should therefore be interpreted as a clinical outcome rather than direct evidence of immune activation. Third, as enrollment has already commenced, adding tissue or blood based translational endpoints would require substantial protocol and ethical amendments and could compromise the consistency of sample collection. Future studies incorporating prospectively defined correlative endpoints are warranted. Fourth, the diversity of fractionation schedules—while a strength of the design—introduces heterogeneity that complicates direct cross cohort interpretation. Finally, the study is conducted in a predominantly Asian population, which may limit generalizability to other ethnic groups.

Despite these limitations, this trial seeks to clarify whether the proposed multimodal strategy is both feasible and clinically promising. The findings are expected to inform the selection of radiotherapy fractionation schedules, support the design of subsequent large−scale randomized studies, and contribute to ongoing efforts to refine neoadjuvant treatment pathways for this common patient population.
